# Phenotypic Analysis of Mutants of Ergosterol Biosynthesis Genes (*ERG3* and *ERG4*) in the Red Yeast *Xanthophyllomyces dendrorhous*

**DOI:** 10.3389/fmicb.2020.01312

**Published:** 2020-06-16

**Authors:** Maximiliano Venegas, Salvador Barahona, Ana María González, Dionisia Sepúlveda, Gustavo E. Zúñiga, Marcelo Baeza, Víctor Cifuentes, Jennifer Alcaíno

**Affiliations:** ^1^Departamento de Ciencias Ecológicas, Facultad de Ciencias, Universidad de Chile, Santiago, Chile; ^2^Centro de Biotecnología, Facultad de Ciencias, Universidad de Chile, Santiago, Chile; ^3^Departamento de Biología, Facultad de Química y Biología, CEDENNA, Universidad de Santiago de Chile, Santiago, Chile

**Keywords:** ergosterol, *ERG3*, *ERG4*, *Xanthophyllomyces dendrorhous*, carotenoids, sterol regulatory element-binding protein

## Abstract

*Xanthophyllomyces dendrorhous* synthesizes astaxanthin, a carotenoid used in aquaculture. Astaxanthin is synthesized from metabolites of the mevalonate pathway, which are also precursors for sterols biosynthesis. The interruption of the *CYP61* gene, which is involved in the synthesis of ergosterol (mutant CBS.*cyp61*^–^), resulted in a phenotype that overproduces carotenoids due to the activation of the SREBP pathway. In this work, we constructed other mutants of ergosterol biosynthesis in this yeast to evaluate whether they have the same phenotype as mutant CBS.*cyp61*^–^. By bioinformatic analysis, the *ERG3* and *ERG4* genes of *X. dendrorhous* were identified, and each gene was deleted in the wild-type strain. Mutants CBS.Δ*erg3* and CBS.Δ*erg4* did not produce ergosterol; CBS.Δ*erg3* primarily accumulated episterol, and CBS.Δ*erg4* primarily accumulated ergosta-5,7,22,24(28)-tetraenol. The transcription levels of the *HMGS* gene of the mevalonate pathway were evaluated by RT-qPCR, which showed a slight increase in CBS.Δ*erg4*, but the transcription levels were still 10-fold lower than in strain CBS.*cyp61*^–^. Both CBS.Δ*erg3* and CBS.Δ*erg4* did not overproduce carotenoids, even though they do not produce ergosterol. Thus, the results of this study indicate that the absence of ergosterol does not activate the SREBP pathway in *X. dendrorhous*, but rather it depends on other alterations in sterol composition.

## Introduction

*Xanthophyllomyces dendrorhous* is a basidiomycete yeast that produces carotenoids, which are natural liposoluble pigments of 40 carbon atoms that exhibit shades ranging from yellow to red (Golubev, 1995) and have antioxidant properties attributed to their structure (Guerin et al., 2003). Among carotenoids, astaxanthin is of biotechnological interest due to its antioxidant capacity, which is the reason why it is currently used in the nutraceutical and cosmetic industries (Johnson, 2003; Higuera-Ciapara et al., 2006). Astaxanthin also has an important economic interest, since it is used in aquaculture as a food supplement.

**FIGURE 1:**
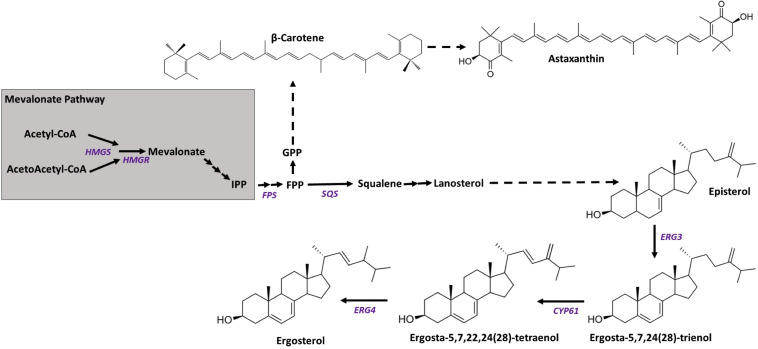
Scheme of the mevalonate pathway and the synthesis of carotenoids and sterols. Arrows represent catalytic steps with the corresponding enzyme-encoding gene studied in this work. Gene names are written in purple, and their GenBank accession numbers are *HMGS* (XDEN_03265), *HMGR* (GenBank: AJ884949), *ERG3* (GenBank: MN930922), *ERG5/CYP61* (GenBank: JX183236), and *ERG4* (GenBank: MN930923). The final steps of ergosterol biosynthesis correspond to steps described in *S. cerevisiae* steroid biosynthesis at the KEGG database (sce00100).

In *X. dendrorhous*, the biosynthesis of astaxanthin begins with the condensation of dimethylallyl-pyrophosphate (DMAPP, C5) and isopentenyl-pyrophosphate (IPP, C5) (Figure 1), which are synthesized through the mevalonate (MVA) pathway, producing geranyl-pyrophosphate (GPP, C10). Then, a second IPP molecule is added, giving rise to farnesyl-pyrophosphate (FPP, C15), which is a substrate for both sterol and carotenoid synthesis. In sterols synthesis, two FPP molecules are condensed, giving squalene, and in the synthesis of carotenoids, FPP is joined to a third molecule of IPP, giving geranylgeranyl-pyrophosphate (GGPP, C20). The condensation of two molecules of GGPP gives phytoene (the first carotenoid of the synthesis pathway), which is transformed into lycopene through four desaturation steps. The cyclization of both ends of lycopene produces β-carotene, and finally, the addition of a keto and a hydroxyl to both terminal rings of β-carotene gives astaxanthin (Loto et al., 2012).

Sterols regulate biological processes and contribute to the cell membrane structure, where they are considered membrane stabilizers (Zhang and Rao, 2010). While cholesterol is the main sterol in mammals, ergosterol plays a key role in fungi. Plants generally have more complex sterol compositions (Schaller, 2003). In the yeast *Saccharomyces cerevisiae*, the biosynthesis of ergosterol has been extensively studied, and the last three steps are catalyzed by enzymes encoded by the *ERG3*, *ERG5*, and *ERG4* genes. The *ERG5* gene is also referred to as *CYP61*, since the gene product belongs to the superfamily of hemoproteins known as cytochrome P450s (P450s). P450s are monooxygenases that are present in all domains of life, and their function is related to oxidative metabolism (Degtyarenko and Archakov, 1993). Two P450s, Cyp51 and Cyp61, are crucial in the synthesis of ergosterol; for this reason, clinical studies against fungal infections have attempted to develop compounds that target P450 enzymes (Parker et al., 2014).

In *S. cerevisiae*, the *ERG3* gene encodes the C-5 sterol desaturase [EC:1.14.19.20], which catalyzes the conversion of episterol to ergosta-5,7,24(28)-trienol using NADPH as a cofactor, a reaction that involves the *cis* removal of hydrogens located in the α position of carbons 5 and 6 of episterol coupled to the reduction of an oxygen molecule. It has been described that the *erg3*^–^ mutation in *Candida glabrata* affects yeast growth, resulting in a phenotype that is not capable of producing ergosterol, thereby accumulating other intermediary sterols (Geber et al., 1995). It has also been described that the *ERG3* gene is essential for growth in *S. cerevisiae* when cultured with fermentable carbon sources, such as glycerol and ethanol (Smith and Parks, 1993). On the other hand, the *ERG4* gene encodes a C-24 sterol reductase [EC:1.3.1.71] that participates in the last step of ergosterol biosynthesis and converts ergosta-5,7,22,24(28)-tetraenol into ergosterol using NADPH as a cofactor. The *S. cerevisiae erg4*^–^ mutant does not produce ergosterol and accumulates the precursor ergosta-5,7,22,24(28)-tetraen-3β-ol. Under the tested conditions, the *erg4*^–^ mutation did not affect cell viability, although it reduced the yeast resistance to different stress conditions and increased its sensitivity to multiple chemicals, thereby reducing its competitive fitness (Zweytick et al., 2000).

In *X. dendrorhous*, two genes controlling the synthesis of ergosterol have been functionally described: *CYP51*/*ERG11* (Leiva et al., 2015) and *CYP61*/*ERG5* (Loto et al., 2012), both of which encode P450 enzymes (Córdova et al., 2017). The *CYP61* gene has been characterized in *S. cerevisiae* as a non-essential gene (Skaggs et al., 1996). Interestingly, the *cyp61*^–^ mutation, in addition to blocking ergosterol biosynthesis, enhanced the production of carotenoids and sterols in different *X. dendrorhous* strains (Loto et al., 2012). Similarly, astaxanthin-overproducing *Phaffia rhodozyma* [the anamorphic state of *X. dendrorhous* (Golubev, 1995)] mutants obtained by random mutagenesis showed a lower ergosterol content compared to the original strains (Miao et al., 2010, 2011).

Recent works revealed that the carotenoid and sterol overproducer phenotype in *X. dendrorhous cyp61*^–^ mutants depends on the sterol regulatory element-binding protein (SREBP) pathway (Gutiérrez et al., 2019; Gómez et al., 2020). SREBPs are a family of transcription factors that regulate the expression of genes controlling the synthesis of sterols and fatty acids. These factors are synthesized as inactive precursor proteins anchored in the endoplasmic reticulum membrane that, depending on the cellular lipid demand, are activated through proteolytic processing (Bien and Espenshade, 2010). An *X. dendrorhous* SREBP homolog encoded by the *SRE1* gene was recently described and shown to be involved in the regulation of carotenoid and sterol synthesis in this yeast (Gutiérrez et al., 2019). Unlike in the wild-type strain, Sre1 is in its active state in the *cyp61*^–^ mutant when cultured under standard laboratory conditions (Gómez et al., 2020). The above results demonstrate that the *cyp61*^–^ mutant provides the conditions that meet the activation of Sre1, which is responsible for the overproduction of carotenoids and sterols in this strain. In support of this statement, the *SRE1* gene deletion in the *cyp61*^–^ mutant reverted the carotenoid- and sterol-overproducing phenotype, reaching levels equivalent to those produced by the wild-type strain. Conversely, the replacement of the *SRE1* gene in the wild-type strain by the gene version that only encodes the active form of Sre1 favors the production of carotenoids and sterols at levels similar to those produced by the *cyp61*^–^ mutant (Gutiérrez et al., 2019). This background suggests that the absence of ergosterol in the *cyp61*^–^ mutants triggers Sre1 activation in *X. dendrorhous*. Then, to move forward to understand what triggers Sre1 activation in *X. dendrorhous*, ergosterol mutants of genes other than *CYP61* were constructed and analyzed in this work. The genes *ERG3* and *ERG4* were selected, as these genes encode enzymes catalyzing the previous and the following step of the one that is catalyzed by the *CYP61* gene product, and because the mutation of these genes in other yeasts were viable and blocked the synthesis of ergosterol.

## Materials and Methods

### Microorganisms, Plasmids and Culture Conditions

The microorganisms and plasmids used or created in this work are listed in Table 1. The CBS 6938 strain of *X. dendrorhous* was used as the wild-type strain, and *E. coli* DH5α was used as a host for plasmid propagation. *X. dendrorhous* strains were cultured at 22°C with constant agitation at 170 rpm in YM medium (0.3% yeast extract, 0.3% malt extract and 0.5% peptone) supplemented with 1% glucose. Yeast transformant selection was performed on YM-agar 1.5% plates supplemented with 20 μg/ml hygromycin B and/or 35 μg/ml zeocin (Sigma, Saint Louis, MI, United States). *E. coli* strains were cultured with constant agitation at 37°C in LB medium (1% tryptone, 0.5% yeast extract and 0.5% NaCl) supplemented with 100 μg/ml ampicillin to select strains carrying plasmids and 40 μl of a 2% solution of X-gal (5-bromo-4-chloro-3-indolyl-β-D-galactopyranoside) for recombinant clone selection (Sambrook and Russell, 2001, #31893). The recombinant clones carrying plasmids with the hygromycin B or zeocin resistance cassette were identified by direct colony PCR with specific primers for each cassette.

**TABLE 1:** Strains and plasmids used and built in this work.

	Genotype or relevant features	Source or references

Strains		
***E. coli***		
DH5α	Used for molecular cloning and plasmid maintenance	Sambrook and Russell, 2001
***X. dendrorhous***		
CBS 6938	ATCC 96594, wild-type strain	ATTC
CBS.*cyp61*^–^	Mutant derived from CBS 6938. The single *CYP61* locus was interrupted by a hygromycin B resistance cassette	Loto et al., 2012
CBS.Δ*erg3*	Mutant derived from CBS 6938. The single *ERG3* locus was replaced by a hygromycin B resistance cassette	This work
CBS.Δ*erg4*	Mutant derived from CBS 6938. The single *ERG4* locus was replaced by a zeocin resistance cassette	This work

**Plasmids**		

pBluescript SK- (pBS)	ColE1 ori; AmpR; cloning vector with blue-white selection	Stratagene
pMN-*hph*	pBS containing at the *Eco*RV site the hygromycin B resistance cassette of 1.8 kb, used for *X. dendrorhous* transformant selection	Niklitschek et al., 2008
pIR-*zeo*	pBS containing at the *Eco*RV site the zeocin resistance cassette of 1.2 kb used for *X. dendrorhous* transformant selection	Loto et al., 2012
pudErg3	pBS containing at the *Eco*RV site the upstream (585 bp) and downstream (496 bp) regions of the *ERG3* ORF	This work
pudErg4	pBS containing at the *Eco*RV site the upstream (668 bp) and downstream (610 bp) regions, with a *Hpa*I site in between, of the *ERG4* ORF	This work
pΔErg3_Hyg	pudErg3 containing the hygromycin B resistance cassette at the *Hpa*I site between the upstream and downstream regions of the *ERG3* gene.	This work
pΔErg4_Zeo	pudErg4 containing a zeocin resistance cassette at the *Hpa*I site between the upstream and downstream regions of the *ERG4* gene	This work

*CBS, Centraalbureau voor Schimmelcultures, Utrecht, Netherlands, ATCC, American Type Culture Collection.*

### Bioinformatic Analyses and Nucleic Acid Extraction

The *ERG3* and *ERG4* genes of *X. dendrorhous* were identified by local tBLASTn search over the genomic and transcriptomic databases from the *X. dendrorhous* strain UCD 67-385 (Baeza et al., 2015) with Geneious R11 using related sequences obtained from the GenBank database as queries. The exon-intron structure of each gene was manually cured by comparing genomic and transcriptomic data. Both gene sequences were uploaded to the GenBank database under the following accession numbers MN930922 and MN930923 for *ERG3* and *ERG4*, respectively. The analysis of the promoter regions of the *HMGS*, *ERG3* and *ERG4* genes was carried out with the JASPAR^1^ platform using the TRANSFAC database (Mathelier et al., 2016). The transcriptional factor SREBF2 was used as a consensus binding site. Protein sequence analyses were performed with programs available at http://www.ebi.ac.uk/interpro (Jones et al., 2014), https://embnet.vital-it.ch/software/TMPRED_form.html (Hofmann and Stoffel, 1993), and https://psort.hgc.jp/form2.html (Nakai and Horton, 1999).

Plasmid DNA was extracted from *E. coli* using the GeneJET Plasmid Miniprep Kit (Thermo Fisher, Waltham, MA, United States), and DNA sequencing was performed at Macrogen, Inc (Seoul, South Korea). The extraction of genomic DNA and total RNA from yeast *X. dendrorhous* was performed by mechanical rupture of a cell pellet obtained from a culture in liquid medium as described previously (Gómez et al., 2020, #70812). The obtained genomic DNA and RNA were stored at −20 and −80°C, respectively, until use.

### Polymerase Chain Reaction (PCR), Reverse Transcription (RT) and Quantitative PCR (qPCR)

All oligonucleotides designed and used in this work were synthesized by Integrated DNA Technologies (Coralville, IA, United States) and are listed in Supplementary Table S1.

To amplify the upstream and downstream fragments of the *ERG3* and *ERG4* genes, PCR analyses were performed using *Pfu* DNA polymerase (Agilent Technologies, Santa Clara, CA, United States). For *E. coli* recombinant clone selection and *X. dendrorhous* transformant confirmation, *Taq* DNA polymerase (Centro de Biotecnología, Facultad de Ciencias, Universidad de Chile, Chile) was used. PCR reactions were performed in a final volume of 25 μl using an Applied Biosystem 2720 thermocycler with the following program for standard PCR: initial denaturation at 95°C for 3 min (or 5 min in case of colony PCR or amplification with *Pfu* DNA polymerase), 35 cycles of denaturation at 94°C for 30 s, alignment of primers at 55°C for 30 s, and elongation at 72°C for 3 to 4 min followed by a final elongation step at 72°C for 10 min, and finally, the reaction was maintained at 4°C until analysis was performed.

Overlap extension PCR (OE-PCR) (Urban et al., 1997) was used to join the upstream and downstream DNA fragments of both studied genes, and the reaction was performed in a final volume of 25 μl with 1 U of *Pfu* DNA polymerase, PCR buffer 1X, each dNTP at 200 μM and 100 ng of each DNA fragment. The following program was used: initial denaturation at 94°C for 1 min, then 10 cycles of denaturation at 94°C for 30 s, DNA alignment at 55°C for 45 s, elongation at 72°C for 90 s, then a final elongation step at 72°C for 10 min, and finally the reaction was kept at 4°C until the next step. Then, the obtained reaction mixture was amplified by a standard PCR analysis with *Pfu* DNA polymerase and the same forward and reverse primers used to amplify the upstream and downstream DNA fragments, respectively.

RT reactions were performed in a final volume of 20 μl. First, 5 μg of total RNA in 11 μl of sterile water was mixed with 1 μl of oligo-dT (25 μM) and 1 μl of dNTPs (at 10 mM each) and incubated at 65°C for 5 min. Then, 1 μl of the enzyme Maxima Reverse Transcriptase (Thermo Fischer, Waltham, MA, United States), 4 μl of RT buffer 5X and 2 μl of DTT (0.1 M) were added. The mixture was incubated at 37°C for 52 min and then left at 70°C for 15 min, enabling it to cool at 4°C.

Real-time qPCR reactions were performed in a Mx3000P real-time PCR system (Agilent, Santa Clara, CA, United States) using Fast EvaGreen qPCR Master Mix (Bio-Rad Laboratories, Hercules, CA, United States). Samples were prepared in a final volume of 20 μl containing 1 μl of cDNA, 0.25 μM of each primer and 10 μl of kit reaction mixture. The efficiency of the primers used was greater than 95%, as determined by standard curves with a correlation coefficient *R*^2^ ≥ 0.99. The obtained Ct (threshold cycle) values were normalized with respect to the corresponding value of the *X. dendrorhous* β-actin gene [GenBank: X89898.1] and expressed as fold change with respect to the control (Livak and Schmittgen, 2001).

### Plasmid Construction and *X. dendrorhous* Transformation

The *ERG3* and *ERG4* genes were replaced by an antibiotic resistance cassette in the *X. dendrorhous* genome by homologous recombination using plasmids pΔErg3_Hyg and pΔErg4_Zeo (Table 1). Plasmids were constructed by joining the upstream (“UP”) and downstream (“DOWN”) regions of each gene of interest by OE-PCR, introducing an *Hpa*I recognition site between them, according to the primer design (primers 5, 6, 15, and 16, Supplementary Table S1). Both regions were PCR-amplified from genomic DNA from *X. dendrorhous* strain CBS 6938, and the OE-PCR product was inserted at the *Eco*RV site of plasmid pBluescript SK to obtain plasmids pudErg3 and pudErg4. Then, each plasmid was digested with *Hpa*I to insert either a hygromycin B or zeocin resistance cassette, which were obtained by *Eco*RV digestion of plasmids pMN-*hph* or pIR-*zeo* (Table 1), respectively.

*Xanthophyllomyces dendrorhous* was transformed by electroporation using a GenePulser Xcell^TM^ (BioRad, Hercules, CA, United States). Electrocompetent cells were prepared from cultures in YM medium at the exponential phase of growth at an OD_600 nm_ of 4–5 (Adrio and Veiga, 1995). Electroporation was performed using 6 μl of transforming DNA (10 μg) employing a 2 mm cuvette and the following conditions: 450 V, 125 μF and 600 Ω. After the pulse, 1 ml of YM was added to the cells, which were incubated for 4 h at 22°C before seeding in YM-agar 1.5% plates with the respective antibiotics to select the transformants.

### Phenotypic Analyses of the Mutant Strains

Phenotypic analysis was performed using at least three biological replicas of each strain, including the mutant strains CBS.Δ*erg3* and CBS.Δ*erg4* and strains CBS.*cyp61*- and CBS 6938 as controls. Strains were cultivated in 100 ml of YM in 250 ml Erlenmeyer flasks at 22°C with constant agitation. Yeast growth was evaluated by the OD_600 nm_ of the culture, which was measured in a UV-vis V-630 spectrophotometer from JASCO. After 120 h of culture (stationary phase of growth), samples were taken to extract carotenoids, ergosterol and total RNA. This time point was selected as major differences in the transcript levels of gene *HMGR* of the MVA pathway, which is a known Sre1 gene target in *X. dendrorhous* (Gutiérrez et al., 2019), were observed when strain CBS.*cyp61*^–^ was compared to the wild-type (Loto et al., 2012).

Extracted carotenoids and sterols were quantified spectrophotometrically and normalized to the dry weight of the yeast. Carotenoids were extracted from cell pellets from 20 ml culture samples disrupted using glass beads and acetone (An et al., 1989) and quantified at 465 nm using an absorption coefficient of A_1%_ = 2,100. Sterols were extracted according to Shang et al. (2006). In brief, cell pellets from 5 ml culture samples were saponified in 16 ml of 60% ethanol and 4 *g* of KOH at 80°C for 2 h. Then, after the mixture was cooled, 5 ml of petroleum ether was added, vortexed for 10 s and centrifuged for 5 min. The supernatant was recovered, and this last step was repeated. Total sterols were quantified at 280 nm using an absorption coefficient of A_1%_ = 11,500.

Carotenoid and sterol compositions were evaluated by reverse-phase liquid chromatography (RP-HPLC) using a LiChrospher RP18 125-4 C-18 column (Merck Millipore, Billerica, MA United States). Carotenoid and sterol samples were run using acetonitrile: methanol: isopropanol (85: 10: 5, v/v/v) and methanol: water (97: 3, v/v), respectively, as the mobile phase with a 1 ml/min flux under isocratic conditions, and the elution spectra were recovered using a diode array detector. Sterols and carotenoids were visualized at the 280 and 474 nm channels, respectively, and compared to standards according to retention time and absorption spectra. The ergosterol standard was acquired from Sigma (Saint Louis, MI, United States).

Sterol identification was performed by a 1100 HPLC Series system equipped with a G1322A degasser and G1311A binary pump. For separation, a reversed-phased Zorbax SB-C18 analytical column (100 mm × 3.0 mm i.d., 5 μm particles) fitted with precolumn Zorbax SB-C18 was used. The mobile phase was prepared from methanol and acetonitrile 30: 70 (v/v). The flow rate was 0.6 ml/min, and 20 μl was injected. All solvents used were filtered through 0.5 ml Sartorius filters and degassed with ultrasounds. The flow rate was set to 0.5 ml/min, and the column temperature was set at 40°C. The mass spectra were acquired in positive mode using electrospray ionization, and analysis of all analytes was carried out in MRM mode. The other operating parameters were as follows: nebulizer gas flow, 3 L/min; drying gas flow, 15 L/min; desolvation line (DL) temperature, 250°C; and heat block temperature, 400°C. All chromatographic data were processed using ChemStation (v A09.03) software and Data Analysis (v 5.3).

## Results

### Isolation and Sequence Analysis of the *ERG3* and *ERG4* Genes From *X. dendrorhous*

The *ERG3* and *ERG4* genes from *X. dendrorhous* were identified by BLAST search over a genomic and transcriptomic databases from the yeast using homologous sequences from other organisms as queries. Once genes were identified, the exon-intron structure of each gene was manually cured by comparing the genomic and transcriptomic data. The *ERG3* gene contains 6 exons of 554, 68, 102, 44, 105, and 114 bp, and the *ERG4* gene has 15 exons of 202, 107, 97, 276, 134, 133, 4, 93, 35, 151, 16, 17, 55, 103, and 35 bp. From the coding sequence, it was deduced that the *ERG3* gene encodes a polypeptide of 328 amino acids with an estimated molecular weight of 38.1 kDa and an isoelectric point (PI) of 7.88. In addition, the ERG3 polypeptide has 26 positively charged and 25 negatively charged residues. The *ERG4* gene encodes a polypeptide of 485 amino acids. This polypeptide is different from the one deposited in the NCBI database, given that our CDS prediction contemplates the CDS of sterol reductase/beta-sheet receptor (CED83197.1) and C-24 sterol reductase (CED83198.1), with an estimated molecular weight of 55.1 kDa and a PI of 9.14. It has 31 negatively charged residues and 43 positively charged residues.

**FIGURE 2:**
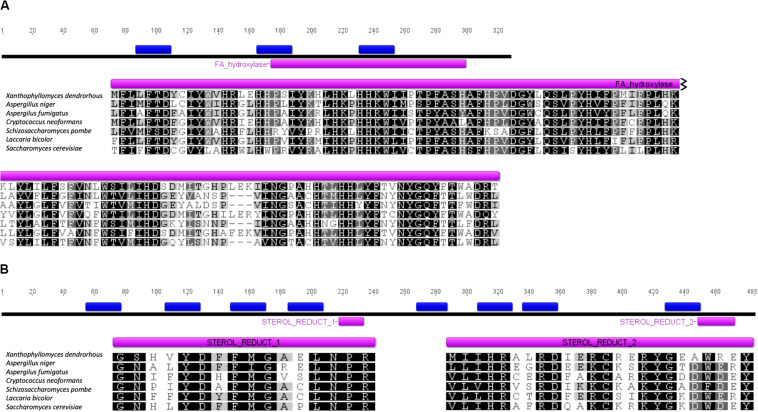
Sequence analysis of the *X. dendrorhous* Erg3 and Erg4 predicted proteins. **(A)** Representation of the deduced Erg3 protein and sequence alignment of the sterol desaturase/sphingolipid hydroxylase, fatty acid hydroxylase superfamily (FA_hydroxylase; COG3000) domain (residues 174 to 299 of the *X. dendrorhous* Erg3 protein). **(B)** Representation of the deduced Erg4 protein and sequence alignment of the two identified sterol reductase (Sterol_Reduct_1 and 2; PS01017 and PS01018) domains (residues 218 to 233 and 449 to 472 of the *X. dendrorhous* Erg4 protein). Predicted transmembrane segments are represented in blue boxes. The UniProt accession numbers (Erg3; Erg4) of sequences used in the alignments are *Aspergillus niger* (A0A100II78_ASPNG; A0A254U2Z2_ASPNG), *Aspergillus fumigatus* (Q2VWQ7_ASPFM; Q4WKA5_ASPFU), *Cryptococcus neoformans* (Q5KNX1_CRYNJ; Q6U7Q6_CRYNH), *Schizosaccharomyces pombe* (ERG31_SCHPO; STS1_SCHPO), *Laccaria bicolor* (B0DA59_LACBS; B0CTW1_LACBS) and *Saccharomyces cerevisiae* (P32353; P25340). Alignments are colored by percentage identity as follows: *black* 100% identity, *gray*>80% identity, *light gray*>60% identity and *white*<60% identity.

The subcellular localization of each protein was predicted using the PSORT II prediction tool. Both proteins are predicted to associate with the endoplasmic reticulum with 34.8% for Erg3p in the K-nn test and 55.6% for Erg4p, which is consistent with their potential function in the biosynthesis of ergosterol at the endoplasmic reticulum (Nishino, 1981). The number of possible transmembrane segments in each of them was also predicted by using the TMHMM Server v. 2.0, where it was observed that Erg3p has 3 transmembrane segments (Figure 2A), while Erg4p has 9 (Figure 2B). Additionally, through the InterPro platform, the presence of conserved domains for both proteins were predicted *in silico*. Erg3p contains 1 domain of the fatty acid hydroxylase superfamily (IPR006694), while for Erg4p, two conserved domains belonging to the family of sterol reductase proteins were observed (IPR018083).

To evaluate the functionality of both identified genes, they were independently replaced by an antibiotic resistance module in the *X. dendrorhous* genome. To this end, recombination modules were constructed and inserted at the *Eco*RV site of plasmid pBluescript II SK- to obtain plasmids pCBS-Δ*erg3* and pCBS-Δ*erg4*, respectively (Table 1). The *ERG3* recombination module contained 585 bp upstream and 538 bp downstream of the gene flanking a hygromycin B resistance cassette, while the *ERG4* recombination module contained 610 bp upstream and 579 bp downstream of the gene and a zeocin resistance cassette between them.

**FIGURE 3:**
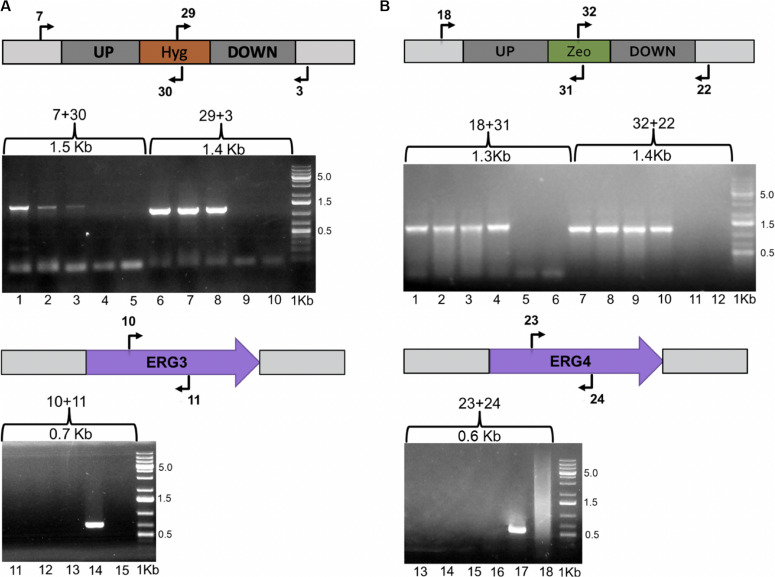
Evaluation of the Δ*erg3* and Δ*erg4* gene mutations. The *X. dendrorhous ERG3* and *ERG4* genes were replaced by hygromycin B and zeocin resistance modules through homologous recombination, respectively, which was confirmed by PCR analysis. Schemes including the primers used in the analysis (Supplementary Table S1) and the expected PCR-product size are indicated in the figure. **(A)** The replacement of the *ERG3* locus by the hygromycin B resistance module was confirmed with primer pairs 7 + 30 and 29 + 3, and the presence of the *ERG3* gene was confirmed with primer pair 10 + 11. Lanes 1, 2, 3, 6, 7, 8, 11, 12, and 13 correspond to the analysis of the three transformants obtained; lanes 4, 9 and 14 to the parental strain CBS 6938, and lanes 5, 10, and 15 to PCR negative controls. **(B)** The replacement of the *ERG4* locus by the zeocin resistance module was confirmed with primer pairs 18 + 31 and 32 + 22, and the presence of the *ERG4* gene was confirmed with the primer pair 23 + 24. Lanes 1, 2, 3, 4, 7, 8, 9, 10, 13, 14, 15, and 16 correspond to the analysis of three transformants; lanes 5, 11 and 17 correspond to the parental strain CBS 6938, and lanes 6, 12 and 18 correspond to PCR negative controls. 1 Kb Plus was used as molecular weight marker 0.5, 1.5, and 5.0 corresponding to the molecular weight sizes in Kb.

Once plasmids pCBS-Δ*erg3* and pCBS-Δ*erg4* were obtained, the wild-type strain CBS 6938 was transformed to replace the respective gene by the antibiotic resistance module through homologous recombination. In this way, three transformants resistant to hygromycin B and four transformants resistant to zeocin were obtained for the *ERG3* and *ERG4* gene mutations, respectively. To confirm the gene replacement events on the obtained transformants, their genomic DNA was extracted, and PCR analyses were performed using an informative set of primers (Figure 3). For the following analysis, a mutant of each gene was randomly selected.

### Evaluation of the Phenotype of the Mutant Strains: Production of Ergosterol and Carotenoids

**FIGURE 4:**
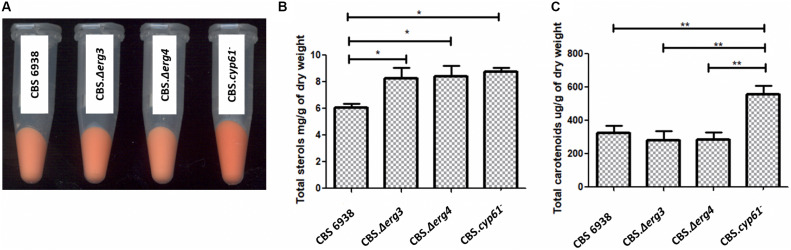
Phenotypic analysis, total sterols and carotenoids between strains CBS 6938, CBS.Δ*erg3*, CBS.Δ*erg4* and CBS.*cyp61*^–^. **(A)** Cell pellets of strains CBS 6938 (parental, wild-type strain), CBS.Δ*erg3*, CBS.Δ*erg4*, and CBS.*cyp*61^–^ obtained from cultures grown on semisolid YM medium incubated at 22°C for 4 days. **(B)** Sterols and **(C)** carotenoids were extracted at the stationary phase of growth cultures (120 h of culture) and quantified at 280 nm and 474 nm, respectively. Values correspond to the average of 3 biological replicas, and the error bars correspond to the standard deviation. Data were normalized with respect to the dry weight of the yeast. *p < 0.05 and **p < 0.01, Student’s *t*-test.

Four strains were analyzed and compared: the wild-type strain CBS 6938 and the mutant strains CBS.Δ*erg3*, CBS.Δ*erg4* and CBS.*cyp61*^–^ (Figure 4A). Only strain CBS.Δ*erg4* showed a different growth curve, reaching a lower OD at the stationary phase of growth (Supplementary Figure S1).

Considering that the genes *ERG3* and *ERG4* are involved in the biosynthesis of ergosterol, total sterols were extracted and quantified (Figure 4B). A significant difference was observed in the total amount of sterols produced by the mutant strains CBS.Δ*erg3* and CBS.Δ*erg4* with respect to the parental strain CBS 6938; both mutants have a higher sterol content than the wild-type strain. Neither strain CBS.Δ*erg3* nor CBS.Δ*erg4* produced ergosterol but rather accumulated other sterols with different retention times (Supplementary Table S2). In addition, to determine the complete profile of sterols in each strain, an LC-MS/MS analysis was performed (Table 2). It was observed that the major compound in strain CBS 6938 corresponds to ergosterol, and it also accumulates other sterols from different levels of the ergosterol synthesis pathway. None of the mutant strains analyzed in this work accumulated ergosterol, and they had a different sterol profile according to each mutation (the affected enzyme). Thus, the main intermediary accumulated in strain CBS.Δ*erg3* corresponded to episterol. In this case, the affected enzyme converts episterol into ergosta-5,7,24(28)-trienol. The strain CBS.Δ*erg4* accumulated ergosta-5,7,22,24(28)-tetraenol since the affected enzyme catalyzes the conversion of this molecule to ergosterol. Finally, the CBS.*cyp61*^–^ strain accumulated ergosta-5,7,24(28)-trienol and episterol as major compounds because the affected enzyme catalyzes the conversion of ergosta-5,7,24(28)-trienol to ergosta-5,7,22,24(28)-tetraenol. In addition, all mutants accumulated upstream sterols, such as lanosterol, zymosterol and fecosterol. In summary, this analysis confirmed that none of the analyzed mutants accumulated ergosterol.

**TABLE 2:** Composition (in relative abundance) of sterols of the mutant and parental strains of *X. dendrorhous* analyzed by HPLC/MS.

	Strain			
Metabolite	CBS 6938	CBS.Δ*erg3*	CBS.Δ*erg4*	CBS.*cyp61*^–^
Ergosterol	36,000 ± 2,160	ND	ND	ND
Ergosta-5,7,22,24(28)-tetraenol	700 ± 42	ND	14,000 ± 980	ND
Ergosta-5,7,24(28)-trienol	800 ± 56	ND	3,200 ± 192	20,000 ± 1,200
Episterol	10,000 ± 600	26,000 ± 1,820	24,000 ± 1,440	18,000 ± 900
Fecosterol	3,000 ± 180	5,300 ± 371	6,300 ± 330	3,000 ± 180
Zymosterol	3,500 ± 210	3,500 ± 210	2,500 ± 150	3,000 ± 180
Lanosterol	3,200 ± 192	14,000 ± 980	14,000 ± 980	13,000 ± 910

*Table shows the mean of the relative abundance of the majority ion of each molecule ± standard deviation of three independent cultures of each strain. ND, not detected.*

Previously, a different color phenotype was observed in *cyp61*^–^ mutants with respect to their parental wild-type strains (Loto et al., 2012); however, this difference was not observed in the CBS.Δ*erg3* or the CBS.Δ*erg4* mutants. To confirm these observations, carotenoids from the analyzed strains were extracted and quantified: only the CBS.*cyp61*^–^ mutant had a significantly higher carotenoid content than the other strains (Figure 4C). Carotenoid composition was also analyzed, and minimal differences were observed among strains (Table 3). These results indicate that even though the CBS.Δ*erg3* and CBS.Δ*erg4* mutants do not produce ergosterol, carotenoid production is not enhanced, as was observed in the *X. dendrorhous cyp61*^–^ mutants, suggesting that is not the absence of ergosterol what enhances carotenoid production but probably the different sterol composition in this mutant would favor carotenoid production.

**TABLE 3:** Composition (in %) of carotenoids of the mutant and parental strains of *X. dendrorhous* analyzed by RP-HPLC.

	Strains			
Metabolite	CBS 6938	CBS.Δ*erg3*	CBS.Δ*erg4*	CBS.*cyp61*^–^
Astaxanthin	70.0 ± 2,0	71.6 ± 0.7	69.7 ± 2.1	76.0 ± 2.4*
Phoenicoxanthin	10.1 ± 1.9	8.6 ± 0.3	13.1 ± 0.6*	13.3 ± 0.6*
β-carotene	7.8 ± 1.4	3.9 ± 0.7**	3.9 ± 0.5**	3.3 ± 0.2**
OH-equinenone	8.8 ± 1.1	6.8 ± 0.8	3.1 ± 0.1**	2.4 ± 0.4**
OH-k-torulene	3.2 ± 0.2	4.6 ± 0.4*	3.3 ± 0.4	5.1 ± 0.7**
Canthaxanthin	4.5 ± 1.0	2.4 ± 0.5*	1.7 ± 0.4*	1.3 ± 0.4*
Equinenone	1.4 ± 0.4	1.1 ± 0.1	1,4 ± 0.1	2.0 ± 0.8
Other Carotenoids	1.0 ± 0.2	1.0 ± 0.1	5.6 ± 0.5**	1.3 ± 0.2
Total percentage	100	100	100	100

Total carotenoids were extracted after 120 h of culture. Table shows the mean ± standard deviation of three independent cultures of each strain. Data were evaluated with Student’s t-test comparing each mutant with the parental strain (*p < 0.05; **p < 0.01).

### Expression Level of the *ERG3*, *ERG4*, and *HMGS* Genes

In a previous work, we observed that *cyp61*^–^ mutants derived from different *X. dendrorhous* wild-type strains had higher transcript levels of the *HMGR* gene, which is involved in the MVA pathway, in relation to the corresponding parental strains (Loto et al., 2012). Transcriptional activation of several genes involved in the MVA pathway and in the synthesis of ergosterol in different organisms is regulated by sterol levels through proteins SREBPs (named Sre1 in several fungi, including *X. dendrorhous*). In general, at low sterol levels, SREBP/Sre1 is activated by proteolytic cleavage, allowing the transcription factor domain to travel to the nucleus and bind to specific DNA sequences, known as sterol regulatory elements (SREs), in the promoter region of target genes to regulate their expression. Conversely, when sterol levels increase, SREBP/Sre1 cleavage is inhibited; therefore, the synthesis of sterols is reduced through a negative feedback mechanism (Hughes et al., 2005). Considering that the *ERG3* and *ERG4* genes encode enzymes involved in ergosterol biosynthesis such that they could be regulated by Sre1, the promoter region of these genes was analyzed to search for potential SREBP/Sre1 binding sites. For comparative purposes, the promoter region of the *HMGS* gene was also analyzed, as it was demonstrated that Sre1 in *X. dendrorhous* binds to this DNA (Gutiérrez et al., 2019). The consensus SRE sequence recognized by the human transcription factor SREBF2 was predicted in the promoter region of the three analyzed genes: *HMGS* has four potential SRE sequences: one located at the [+] strand and three at the [−] strand, *ERG3* has two potential SREs at the [+] strand and *ERG4* has one potential SRE at the [−] strand (Supplementary Table S3).

**FIGURE 5:**
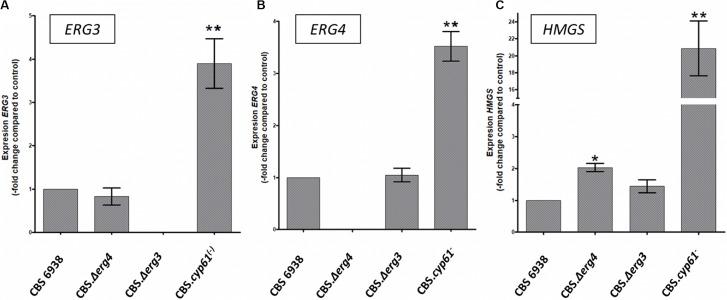
Transcript levels of the *HMGS*, *ERG3* and *ERG4* genes from the *X. dendrorhous* strains studied in this work. Relative transcript levels of genes **(A)** *ERG3*, **(B)** *ERG4*, and **(C)** *HMGS* were determined by RT-qPCR after 120 h of culture and normalized to the housekeeping gene encoding β-actin (GenBank: X89898.1). Values are the average ± standard deviation of three independent cultures (**p < 0.01, *p < 0.05; Student’s *t*-test).

To obtain further insight, the relative transcript levels of genes *ERG3*, *ERG4* and *HMGS* were evaluated by RT-qPCR after 120 h of culture in the four *X. dendrorhous* strains studied in this work (Figure 5). Interestingly, transcript levels of the three genes were higher in strain CBS.*cyp61*^–^. Considering this result and that potential SRE sequences were identified in their promoter regions, their expression at the transcriptional level could indeed be regulated by Sre1, which is in its active form in strain CBS.*cyp61*^–^. Although mutants CBS.Δ*erg3* and CBS.Δ*erg4* do not produce ergosterol, in general, the transcript levels of the three genes did not change with respect to the wild-type strain. The only exception was the *HMGS* gene in strain CBS.Δ*erg4*, which showed higher transcript levels than the wild-type strain; however, this increment was only approximately 2-fold in contrast to 20-fold observed in strain CBS.*cyp61*^–^. In addition, the *ERG3* and *ERG4* transcripts were not detected in the respective mutant strains, confirming that these mutants do not express these genes.

## Discussion

The genetic control of ergosterol biosynthesis has been widely studied in *S. cerevisiae* (Bard et al., 1996; Mo et al., 2002; Shobayashi et al., 2005), whereas in *X. dendrorhous*, only the *CYP61/ERG5* (Loto et al., 2012) and *CYP51/ERG11* (Leiva et al., 2015) genes have been functionally described. In this work, two additional genes were described: *ERG3* and *ERG4*, encoding enzymes involved in the late steps of ergosterol biosynthesis. From bioinformatic analyses, it was predicted that the encoded proteins would be located at the endoplasmic reticulum, which is consistent with their function as in *S. cerevisiae*, the ergosterol biosynthesis ends in this organelle (Nishino, 1981). Additionally, it was deduced that the *X. dendrorhous* proteins Erg3 (328 aa) and Erg4 (485 aa) have a similar size to that described in *S. cerevisiae*, 365 and 473 aa, respectively (Smith and Parks, 1993; Zweytick et al., 2000). The *X. dendrorhous* proteins have the corresponding characteristic domains (Figure 1). Protein Erg4 has two steroid reductase domains that are present in the Erg4 and Erg24 proteins in yeast (Lai et al., 1994; Holmer et al., 1998), as both Erg4 and Erg24 are responsible for reducing double bonds in molecules. In Erg3, the hydroxylase family domain was identified; this family includes the Erg3 and Erg25 proteins of ergosterol biosynthesis. These results support that the gene product of the genes identified in this work could indeed be involved in the ergosterol biosynthesis pathway in *X. dendrorhous*.

High-performance liquid chromatography (HPLC) is a standard method for separating the major sterols in an extract (Knittelfelder and Kohlwein, 2017). In this work, a reproducible and accurate LC-MS/MS technique was used. Besides ergosterol, the other resulting signals presented a good resolution, indicating the suitability of this methodology to assess sterol profiles. Furthermore, this methodology is faster than the widely used gas liquid chromatography (GLC) analysis and operates under milder column temperatures and non-destructive detection conditions (Madla et al., 2012). The employed method proved to be a useful tool for rapidly determining sterol profile, compared with other chromatographic methodologies and sample preparation protocols. LC-MS/MS analysis was performed with little sample purification, avoiding losses of those molecules in low concentrations. As has been reported in other organisms (Geber et al., 1995; Zweytick et al., 2000), the genes studied in this work were not essential for *X. dendrorhous* viability. However, growth was slightly reduced in strain CBS.Δ*erg4*, which could be due to the sterols accumulated in this mutant or another kind of interaction of this gene with the metabolism of the yeast. As strain CBS.Δ*erg4* accumulates a larger amount of ergosta-5,7,22,24(28)-tetraenol than the wild-type (metabolite that was not detected in the other strains), it is possible that this intermediary could be affecting the yeast growth, but further studies are required to support this idea. It was confirmed that the identified *ERG3* and *ERG4* genes are indeed involved in the biosynthesis of ergosterol, since mutants of these genes were not able to produce ergosterol, but they accumulate other sterols. Strain CBS.Δ*erg3* mainly accumulates episterol and strain CBS.Δ*erg4* mainly accumulates ergosta-5,7,22,24(28)-tetraenol, similar to what has been described in homologous *S. cerevisiae* mutants (Geber et al., 1995; Zweytick et al., 2000). By the approximation used in this work, it was observed that strain CBS.*cyp61*^–^ mainly accumulates ergosta-5,7,24(28)-trienol.

The production of sterols increased in both mutants constructed in this work compared to the wild-type (Figure 4B), suggesting an ergosterol-mediated regulatory feedback mechanism to regulate sterol biosynthesis in *X. dendrorhous*, such as the SREBP pathway (Hughes et al., 2005). However, unlike in strain CBS.*cyp61*^–^, the production of carotenoids was not affected in the CBS.Δ*erg3* and CBS.Δ*erg4* mutants compared to the parental strain, nor was their content, nor was their composition (Figure 4C). These results support that it is not the absence of ergosterol that enhances carotenoid production in *X. dendrorhous*, but rather, what favors carotenogenesis in this yeast is the different sterol composition that the *cyp61*^–^ mutants have. This finding suggests that the activation of the SREBP pathway in *X. dendrorhous* is not mainly due to the absence of ergosterol, but it is due to changes in the composition of sterols in the membrane, as was evidenced in *S. pombe* (Hughes et al., 2007). It is possible that ergosta-5,7,24(28)-trienol could play a role in the activation of the SREBP pathway in *X. dendrorhous*, as this was the main sterol accumulated in strain CBS.*cyp61*^–^, but further studies are required.

The step controlled by the *HMGS* gene has been reported as an important regulatory step of the MVA pathway; for example, the overexpression of this gene together with other genetic modifications favored the production of isoprenoid compounds in *S. cerevisiae* (Bröker et al., 2018). In previous works, we demonstrated that the *HMGS* gene is a Sre1 gene target in *X. dendrorhous* (Gutiérrez et al., 2019; Gómez et al., 2020). In this work, the relative transcript levels of the *HMGS* gene were determined, and no differences were observed between the parental strain and strain CBS.Δ*erg3*. However, a 2-fold increase was observed in strain CBS.Δ*erg4* in relation to the parental strain. Even though this increment was not as high as the increment observed in strain CBS.*cyp61*^–^ (Figure 5C), it is interesting to note that strain CBS.Δ*erg4* follows strain CBS.*cyp61*^–^ regarding ergosta-5,7,24(28)-trienol content and that the wild-type strain is in third place in this aspect. Then, it is possible to speculate or suggest that the ergosta-5,7,24(28)-trienol content could be somehow related to Sre1 activation in *X. dendrorhous*: high levels of this sterol (as in strain CBS.*cyp61*^–^) would promote Sre1 activation, middle levels of this sterol (as in strain CBS.Δ*erg4*) would promote some level of Sre1 activation and low levels of this sterol (as in the wild-type strain) would allow having a basal Sre1 activation level. This speculation is supported by western blot analysis of proteins extracted from the wild-type strain cultured under standard conditions (Gómez et al., 2020), where a weak band coinciding with the size of the Sre1 active form was observed, indicating that there is indeed a basal level of Sre1 proteolytic activation in the wild-type strain. However, further studies are required to support this possibility.

In summary, in this work, two genes, *ERG3* and *ERG4*, involved in ergosterol biosynthesis in *X. dendrorhous* were identified and functionally described. Deletion of both genes avoided ergosterol production but exhibited a wild-type color phenotype, unlike the *cyp61*^–^ ergosterol mutant, which overproduces carotenoids. The results presented in this study support that is not the absence of ergosterol what enhances carotenoid production through the activation of the SREBP pathway in *X. dendrorhous*, but rather this phenotype depends on the altered sterol composition observed in the *cyp61*^–^ mutant.

## Conclusion

The deletion of the *ERG3* and *ERG4* genes in *X. dendrorhous* prevents the synthesis of ergosterol and mutants Δ*erg3* and Δ*erg4* accumulate episterol and ergosta-5,7,22,24(28)-tetraenol, respectively. The phenotypic analysis results (production of sterols and carotenoids, and transcript levels of the studied genes), strongly suggest the SREBP pathway is activated in the mutant *cyp61*^–^, but not in the mutants constructed in this work (Δ*erg3* and Δ*erg4*) as sterols and carotenoids levels (and transcript levels) are not affected in the same way. The activation of the SREBP pathway in *X. dendrorhous* could be somehow related to sterol composition, rather than to the absence of ergosterol in the strains.

## Data Availability Statement

The datasets presented in this study can be found in online repositories. The names of the repository/repositories and accession number(s) can be found in the article/Supplementary Material.

## Author Contributions

All authors contributed significantly to the work and agreement with the content of the manuscript. MV, GZ, and AG performed the experiments. SB, DS, MB, VC, and JA provided strategic inputs. MV, GZ, and JA wrote the manuscript.

## Conflict of Interest

The authors declare that the research was conducted in the absence of any commercial or financial relationships that could be construed as a potential conflict of interest.

## Footnotes

1 http://jaspar.genereg.net
