# Supplementary material for: Phenotypic Analysis of Mutants of Ergosterol Biosynthesis Genes (ERG3 and ERG4) in the Red Yeast Xanthophyllomyces dendrorhous
Source: Front Microbiol. 2020 Jun 16;11:1312. doi: 10.3389/fmicb.2020.01312 (PMC7309136; doi:10.3389/fmicb.2020.01312)
Supplement: Supplementary file 1 [file Table_1.docx]

Supplementary Material

# Supplementary Figures and Tables

## Supplementary Table

### Supplementary Table 1. Primers used in this work.

| **N°** | **Name** | **Sequence (5´- 3´)** | **Orientation** |
| --- | --- | --- | --- |
| **1** | Fw_UP1 ERG3 | CGCCGGCCTTGTGGATTTC | **F** |
| **2** | Fw_UP3 ERG3 | CCAACAGCACAGCATCGTG | **F** |
| **3** | Rv_Down1 ERG3 | CCCTTTGTCTCGCCATCTC | **R** |
| **4** | Rv_Down3 ERG3 | CGCCATCTCATTCCCTTAGAC | **R** |
| **5** | Fw_Down ERG3 | CTGGCTTTACGTTAACCTTCCTCCTCTGGTGCTCC | **F** |
| **6** | Rv_UP ERG3 | GAGGAGGAAGGTTAACGTAAAGCCAGGGCTGTGTCG | **R** |
| **7** | Fw_UP2 ERG3 | CGCGTGATTGATTCGTCAC | **F** |
| **8** | RV_Down2 ERG3 | GTC TCCCTAAGCGTGTGGT | **R** |
| **9** | Fw_UP1 ERG3 | CGCCGGCCTTGTGGATTTC | **F** |
| **10** | FW_ERG3C | CCGACTTCTCCCTGGGTGTCC | **F** |
| **11** | RV_ERG3C | CAACCGGGTGGAACGCGTG | **R** |
| **12** | Fw_UpM ERG3 | GCTTCTCTTCTTCCGCCA | **F** |
| **13** | Rv_DownM ERG3 | GAGCAGCAGACAAGGACCG | **R** |
| **14** | Fw_UP1 ERG4 | CCCGTCACCGGTCTAGCCAAC | **F** |
| **15** | Rv_UP ERG4 | TCGGTGAACGGTTAACGTTTGCCTGCTTGCGCTGCC | **R** |
| **16** | Fw_Down ERG4 | GCAGGCAAACGTTAACCGTTCACCGATGCGGTCGAGAC | **F** |
| **17** | Rv_Down1 ERG4 | GTACAGCCTTGCGGCGTTCGAC | **R** |
| **18** | Fw_UP2 ERG4 | GCGCCAACGAACCTTCTCGAC | **F** |
| **19** | Rv_Down2 ERG4 | CCTTCACGGTGGCCCCAAC | **R** |
| **20** | Fw_UP3 ERG4 | CCCCAATGCCTGGGTCAGACC | **F** |
| **21** | Rv_Down3 ERG4 | CGGTCACGAGCTTGAAACTGCC | **R** |
| **22** | Rv_Down4 ERG4 | CACTTCTGCCGTCACTGGC | **R** |
| **23** | FW_ERG4C | CCGCCGGGATAGTCCACTAC | **F** |
| **24** | RV_ERG4C | CGTAGTGGGGATCATCTCCTC | **R** |
| **25** | PEFForEV | GATATCGGCTCATCAGCCGACAGTT | **F** |
| **26** | pEFrev0 | TTTGAAGCTGTTCGAGATAG | **R** |
| **27** | gpdTF | ACGGTTCTCTCCAAACCCTC | **F** |
| **28** | P.TEFR | GGTGAAGCTGTTCGAGATAGA | **R** |
| **29** | H-out.F | CTCGATGAGCTGATGCTTTG | **F** |
| **30** | H-out.R | CACTGGCAAACTGTGATGGA | **R** |
| **31** | Zeo.R | GTGACCCTGTTCATCAGCG | **R** |
| **32** | Zeo.F | GACTTCGTGGAGGACGACTT | **F** |
| **33** | mactR-RT | TCACCAACGTAGGAGTCCTT | **R** |
| **34** | mactF-RT | CCGCCCTCGTGATTGATAAC | **F** |
| **35** | FW1_ERG3Q | CTCCATTCGCCTCCCACG | **F** |
| **36** | FW2_ ERG3Q | CGGTTGACGGGTATCTCCAA | **F** |
| **37** | RV1_ERG3Q | GGCCGGTCCGTTAATGATCT | **R** |
| **38** | FW1_ERG4Q | GCTTCTTACTTCCGGCTGGT | **F** |
| **39** | FW2_ERG4Q | GACTTGGGGTCTCTCTACCG | **F** |
| **40** | RV1_ERG4Q | CCTTTCCGTACTTGGCTGCAC | **R** |
