# Supplementary material for: Phenotypic Analysis of Mutants of Ergosterol Biosynthesis Genes (ERG3 and ERG4) in the Red Yeast Xanthophyllomyces dendrorhous
Source: Front Microbiol. 2020 Jun 16;11:1312. doi: 10.3389/fmicb.2020.01312 (PMC7309136; doi:10.3389/fmicb.2020.01312)
Supplement: Supplementary file 2 [file Table_2.docx]

Supplementary Material

# Supplementary Figures and Tables

### Supplementary Table 2. Composition (in %) of Sterols of the mutant and parental strains of *X. dendrorhous* analyzed by RP-HPLC.

|  | Strain | | | |
| --- | --- | --- | --- | --- |
| Metabolite | **CBS 6938** | **CBS.Δ*erg3*** | **CBS.Δ*erg4*** | **CBS.*cyp61^-^*** |
| Ergosterol, peak at 14 min | 100 ± 0.0 | ND | ND | ND |
| Peak at 9 min | ND | 100 ± 0.0 | ND | ND |
| Peak at 8 min | ND | ND | 100 ± 0.0 | ND |
| Peak at 12 min | ND | ND | ND | 25 ± 1 |
| Peak at 16 min | ND | ND | ND | 75 ± 4 |
| Total | 100 | 100 | 100 | 100 |
| Sterols were extracted after 120 hours of culture. Table shows the mean ± standard deviation of three independent cultures of each strain. Peaks correspond to metabolites having a sterol spectrum, which were observed in chromatograms after approximately 8, 9, 12 and 16 min of retention time, respectively. | | | | |
