# Supplementary material for: Phenotypic Analysis of Mutants of Ergosterol Biosynthesis Genes (ERG3 and ERG4) in the Red Yeast Xanthophyllomyces dendrorhous
Source: Front Microbiol. 2020 Jun 16;11:1312. doi: 10.3389/fmicb.2020.01312 (PMC7309136; doi:10.3389/fmicb.2020.01312)
Supplement: Supplementary file 3 [file Table_3.DOCX]

Supplementary Material

# Supplementary Figures and Tables

### Supplementary Table 3. SRE sequences predicted in the promoter regions of the *HMGS*, *ERG3* and *ERG4* genes.

| **Name** | **Start (bp)** | **End (bp)** | **Strand** | **Predicted Sequence** |
| --- | --- | --- | --- | --- |
| **HMGS_1** | -769 | -778 | - | gtggtgttac |
| **HMGS_2** | -5 | -14 | - | atgggacgat |
| **HMGS_3** | -876 | -885 | - | atgggacgat |
| **HMGS_4** | -596 | -605 | + | atgagcggat |
| **ERG3_1** | -691 | -682 | + | ctggggtggc |
| **ERG3_2** | -900 | -891 | + | atagagtgac |
| **ERG4_1** | -541 | -532 | - | ctggtctgac |
