# Supplementary material for: Phenotypic Analysis of Mutants of Ergosterol Biosynthesis Genes (ERG3 and ERG4) in the Red Yeast Xanthophyllomyces dendrorhous
Source: Front Microbiol. 2020 Jun 16;11:1312. doi: 10.3389/fmicb.2020.01312 (PMC7309136; doi:10.3389/fmicb.2020.01312)
Supplement: Supplementary file 4 [file Data_Sheet_1.DOCX]

Supplementary Material

# Supplementary Figures and Tables

## Supplementary Figures


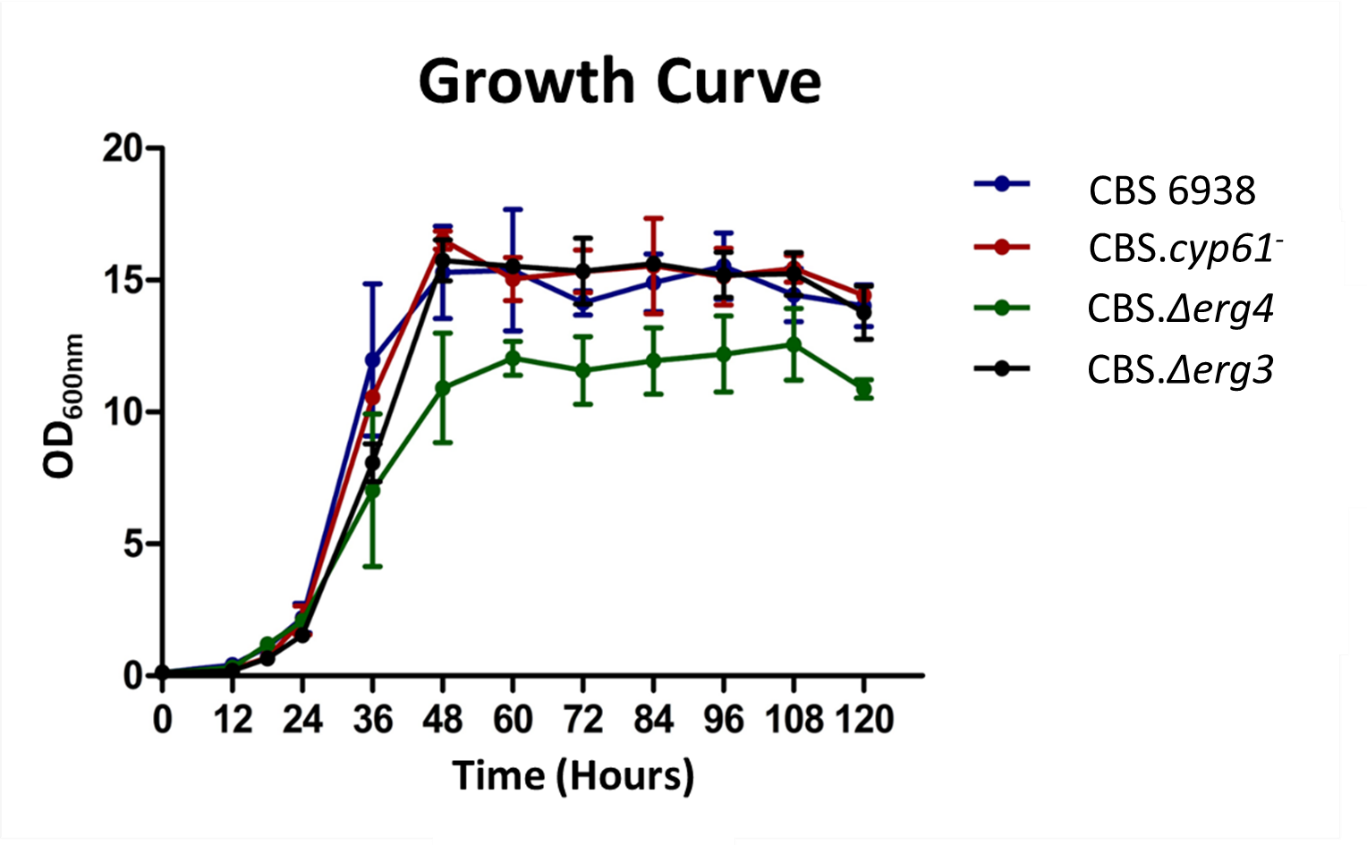


**Supplementary Figure 1.** **Growth curves of strains CBS 6938, CBS.*cyp61^-^*, CBS.*∆erg3* and CBS.*∆erg4*.** Strains were cultured at 22 °C in YM medium with constant agitation. Data are the mean ± standard deviation of three biological replicates. Samples were taken after 120 hours of culture for further analysis (carotenoid, sterol and RNA extraction, according to the corresponding analysis).
